# Brazilian and Mexican experiences in the study of incipient domestication

**DOI:** 10.1186/1746-4269-10-33

**Published:** 2014-04-02

**Authors:** Ernani Machado de Freitas Lins Neto, Nivaldo Peroni, Alejandro Casas, Fabiola Parra, Xitlali Aguirre, Susana Guillén, Ulysses Paulino Albuquerque

**Affiliations:** 1 Department of Sciences of Nature at Universidade Federal do Vale do São Francisco, Campus Senhor do Bonfim, Bahia, Brazil; 2 Department of Ecology and Zoology at Universidade Federal de Santa Catarina, Florianópolis, Santa Catarina, Brazil; 3 Centro de Investigaciones en Ecosistemas at Universidad Nacional Autónoma de México, Campus Morelia-Michoacán, Michoacán, Mexico; 4 Department of Biology, Laboratory of Applied and Theoretical Ethnobiology (LEA) at Universidade Federal Rural de Pernambuco, Recife, Pernambuco, Brazil

**Keywords:** Biodiversity conservation, Crop evolution, Domestication, Ethnobotany, Incipient domestication, Plant management

## Abstract

**Background:**

Studies of domestication enables a better understanding of human cultures, landscape changes according to peoples’ purposes, and evolutionary consequences of human actions on biodiversity. This review aimed at discussing concepts, hypotheses, and current trends in studies of domestication of plants, using examples of cases studied in regions of Mesoamerica and Brazil. We analyzed trends of ethnobiological studies contributing to document processes of domestication and to establish criteria for biodiversity conservation based on traditional ecological knowledge.

**Methods:**

Based on reviewing our own and other authors’ studies we analyzed management patterns and evolutionary trends associated to domestication occurring at plant populations and landscape levels. Particularly, we systematized information documenting: ethnobotanical aspects about plant management and artificial selection mechanisms, morphological consequences of plant management, population genetics of wild and managed plant populations, trends of change in reproduction systems of plants associated to management, and other ecological and physiological aspects influenced by management and domestication.

**Results:**

Based on the analysis of study cases of 20 native species of herbs, shrubs and trees we identified similar criteria of artificial selection in different cultural contexts of Mexico and Brazil. Similar evolutionary trends were also identified in morphology (selection in favor of gigantism of useful and correlated parts); organoleptic characteristics such as taste, toxicity, color, texture; reproductive biology, mainly breeding system, phenological changes, and population genetics aspects, maintenance or increasing of genetic diversity in managed populations, high gene flow with wild relatives and low structure maintained by artificial selection. Our review is a first attempt to unify research methods for analyzing a high diversity of processes. Further research should emphasize deeper analyses of contrasting and diverse cultural and ecological contexts for a better understanding of evolution under incipient processes of domestication.

**Conclusion:**

Higher research effort is particularly required in Brazil, where studies on this topic are scarcer than in Mexico but where diversity of human cultures managing their also high plant resources diversity offer high potential for documenting the diversity of mechanisms of artificial selection and evolutionary trends. Comparisons and evaluations of incipient domestication in the regions studied as well as the Andean area would significantly contribute to understanding origins and diffusion of the experience of managing and domesticating plants.

## Background

Nearly 11,000 years ago, humans started to domesticate plants and animals in the area known as the Fertile Crescent, in southwestern Asia [1]. Cereals, such as barley (*Hordeum vulgare* L.) and wheat (*Triticum* spp.), and legumes, such as lentils (*Lens culinaris* Medik.) and peas (*Pisum sativum* L.), were among the first crop plant species [2-6]. Later on, plant agriculture and domestication were developed in other areas of the World. China, Southeastern Asia, and Sub-Sahara African regions were other important areas in the Old World [1,3], whereas Mesoamerica and the Andean regions have been recognized as the main centers of domestication in the New World [1-6]. In the Neotropics, the oldest records of domestication of plants are remains of *Cucurbita* approximately 10,000 years ago [7]. In Mesoamerica and the Andean regions, the archaeologists documented that prehistoric cultures managed broad spectra of plant resources, including members of the Poaceae, Fabaceae, Euphorbiaceae, Araceae, Solanaceae, and Cactaceae, as well as numerous species of fruit trees [2,3,8]. In Mesoamerica, plants such as maize (*Zea mays* L.), beans (*Phaseolus* sp.) and squashes and pumpkins (*Curcubita* spp.), as well as chili peppers (*Capsicum* spp.) were domesticated while the multi crop-growing system, known in the region as ‘milpa’ was developed [9]. In the Andean area potatoes and several species of tubers were particularly important, as well as quinoa (*Chenopodium quinoa*), kiwicha (*Amaranths caudatus*), squashes (*Cucurbita maxima*) and several species of beans in the artificial ecosystems called ‘chacra’ [1]. The Amazonian region has been proposed as an area where numerous native plant species were domesticated, which was probably influenced by both Andean and Mesoamerican experiences of agriculture [3]. However, more research is needed to compare patterns of plant management and domestication among the three regions. *Manihot esculenta* is among the most representative crops of the Amazonian region [10], but Clement et al. [11] have reported that at least 138 native plant species of Brazil currently show signs of domestication. In addition, several studies [11-15] have reported nearly 180 plant Brazilian native species under some management type.

Artificial selection is generally practiced with the intention to favor the frequency increasing of desirable individuals (species or phenotypes of particular species) in populations [16-18]. Such process involves the human recognition that (1) plant populations show variable attributes, (2) people value differently the recognized variants and (3) they favor the survival and reproduction (fitness) of particular features that are positive to humans [17,18]. Selective pressures may occur at different intensities and determine proportional extent of modifications of genetic structure and phenotypic patterns of populations [6,13] and consequently evolutionary divergence among managed and unmanaged populations. Even in advanced domestication processes, a wide range of states of plants depending on humans for survival and reproduction can be identified [3,14].

The process of domestication has been analyzed at both population and landscape levels [19-22]. From these perspectives, selection by humans may favor not only variants of a single species, but also the presence and increasing numbers of particular groups of species in a biotic community. Therefore, human activities modeling the composition of both plant populations and communities are relevant for understanding domestication from a broader perspective: the management and domestication of landscapes. Plant management commonly involves domestication at early stages or incipient domestication, which is of special importance for understanding early forms of management and origins of agriculture, but which has been relatively little studied. Most studies on on-going domestication processes in the New World have been conducted in Mesoamerica and the Andean area [4], but more recently several studies have been conducted in Brazil and it is possible to start a comparison of patterns among regions; in turn this information will allow comparing domestication patterns among other regions of the New World and other continents. Such comparisons are of theoretical value for testing hypotheses about environmental and human cultural contexts influencing starting of management and domestication which are relevant for understanding the why of origins of agriculture, which is currently a topic of academic controversy.

Our study aimed at analyzing and discussing methods, results, concepts and theories on the process of incipient domestication derived from studies in Mexico and Brazil in order to examine particular management patterns and evolutionary trends of both species and landscapes under domestication in both regions. We particularly analyzed information from ethnobotanical, ecological, and evolutionary studies of the processes of domestication which provide valuable data to define criteria for biodiversity conservation based on traditional ecological knowledge and technologies. We aspire to contribute to a better understanding of the evolutionary processes derived from interactions between people, plant species, and landscapes and to identify methods and priorities of research for a deeper understanding of the human experience of domesticating elements and systems of territories.

## Incipient domestication: concepts and theories

The emergence of agriculture was one of the main revolutionary processes in the history of humanity and studying it has, therefore, motivated research and theories that search for explanations about where, when, how, and why it originated. It has been generally assumed that before food production systems were adopted, human populations accumulated extensive knowledge on plant species that allow them to identify and make use of the different properties of resources. Vast ethnobiological information throughout the world has demonstrated that even after thousands of years that agriculture has been the predominant way of obtaining likelihoods for subsistence, gathering and incipient management of numerous plant species continue being important forms of interaction between people and plants [23]. Traditional knowledge and perception of variation is a necessary condition for favoring some variants instead of others, and this practice is the general principle of artificial selection [24]. Domestication is viewed as an evolutionary process influenced by humans [3] mainly through artificial selection, and this is a crucial interaction between humans and plants and other organisms under domestication.

The earliest author crediting human action as promoter of variation and change in plants and animals was Charles Darwin, firstly in his The Origin of Species [23] and then in The Variation of Animals and Plants under Domestication [24]. Throughout the 20^th^ Century and until present, the concept of domestication has been continually constructed mainly based on the development of archaeological, ethnobotanical, ecological, and genetic studies. Currently, domestication is defined as a process through which humans determine changes in the genetic structure of plant populations in order to favor the frequency of phenotypes and genotypes that are advantageous for humans and their social and cultural life [25,26]. Criteria of humans for artificial selection are based on the cultural values of plants and plant variants considered as resources for satisfying human needs. It has also been suggested that domestication is a co-evolutionary process, determined by management and human selection (conscious or unconscious) of sets of species (biotic communities) and/or individuals of species populations. This process on one hand may favor particular phenotypes composing populations and species composing biotic communities [11,27]. On the other hand, the process of domestication has influenced significant changes of human societies and cultures.

Domestication is recognized to be a continuous process that may occur on wild managed plant populations as well as in fully domesticated plant stands which are completely dependent on humans to survive and reproduce [3,6,11]. In areas where wild relatives of crops and the domesticated organisms coexist it is possible to identify continuous gradients of states or degrees of dependence of plant fitness according to the types of human actions [3,6]. Those plants that can be propagated and managed by people, but not necessarily depend on them for completing their life cycle are called by some authors semi-domesticated or incipient domesticates [6]. Incipiently domesticated plants are those that are in early stages of domestication, with relatively low phenotypic and genetic differentiation compared with their wild relatives. Clement [28] has claimed a distinction between species in incipient state of domestication and those that are semi-domesticated. According to this author, incipient domesticated plants exhibit phenotypic variation within the range normally found in wild populations, whereas semi-domesticated plants are characterized by greater phenotypic variations than their wild ancestors, including the emergence of new characteristics [28]. However, plant populations of plant species in the wild and at initial stages of domestication may show patterns of high morphological variation associated to natural selection and therefore, other additional indicators are needed to arrive to a conclusion about the initial, intermediate or advanced degrees of domestication of plant populations. The fact is that variation in plant populations may diverge by both natural and cultural processes and in all studies of domestication it is necessary to understand which aspects are influenced by natural factors and which ones by human culture. In addition, it is necessary to have in mind that natural and human cultural processes act on populations’ divergence continually and, consequently, a continuum of variation is the most common condition found. Therefore, more precise typologies for systematizing the degrees of variation between wild and managed populations are still needed. In all concepts of domestication, artificial selection is considered as the main evolutionary force, which is in turn influenced by cultural and ecological factors, as well as the amount of gene flow among wild and domesticated relatives. Studying integrally all these factors is necessary to understand how domestication occurs.

Some authors identify plant populations that have incidentally co-evolved with crop plant species (e.g. weeds), some of them having progressed through landraces and then to modern cultivars [28]. According to Clement [28], weeds are plant populations adapted to disturbed habitats, possibly experiencing changes in their genetic structure resulting from ecosystem changes determined by humans although, in most cases, without direct human selection and management. Landraces populations of semi-domesticated or fully domesticated plants display high phenotypic and genetic variation in particular geographic areas. In other extreme, modern cultivars have reduced genetic variation because of the high selective pressure and modifications made to better adapt them to intensive monocultures [19,26,29-31].

Domestication is an evolutionary process that frequently occurs gradually, but some vegetatively propagated plants may be ‘immediately’ domesticated [3]. The interactions between people and plants start in their wild environment. Gathering has been considered for long time as a ‘harvest of nature’, but nowadays numerous ethnobotanical studies have documented that this activity may involve social agreements, special tools, and strategies with different complexity [17]. Interactions become more complex with protected, enhanced or cultivated plants, and even more with plants involving different levels of artificial selection and domestication degrees [17,22]. Studying plant species in incipient and advanced stages of domestication make possible to analyze it as an evolutionary continuum of intensity of management and artificial selection, especially in areas where managed and wild populations coexist. In these areas it is possible to verify gene flow between wild and domesticated populations, their influence in maintaining local diversity, and the influence of natural and artificial selection on their genetic structure. But this is also possible among populations of plant species under incipient stages of domestication, which offers the opportunity to analyze how human management of plants could be in the early stages of agriculture.

## Management types and their influence in processes of incipient domestication of plants: Mexican and Brazilian study cases

### Mexican cases

The Mesoamerican region is one of the main settings of domestication of plants in the world [1-3,5,16,32,33], and important research projects have been and are still being developed in that area to understand cultural and biological principles involved in the process. These researches provide insight into factors that originated agriculture and mechanisms of evolution under domestication [9,23,26]. According to [34], studies on management forms of plant populations and communities by traditional cultures allows analyzing processes of domestication since it has measurable results. It is possible to investigate cultural aspects of artificial selection, management methods involved and to quantify the effects of such practices on biological variables of plant populations.

Studies in Mesoamerica have allowed the identification and characterization of three main types of plant population management strategies by traditional communities: gathering, incipient management, and cultivation of domesticated plants or agriculture. It is also worth noting that this gradient can be observed in hundreds of species of dozens of plant families. Some in depth studies have been conducted with members of the families Agavaceae [20], Bombacaceae [35], Cactaceae [21,36-41], Malpighiaceae [42], Solanaceae [43-50], Curcubitaceae [51,52] and Fabaceae [53-62] among others, and some general management patterns and evolutionary trends of managed plants have been identified in the Mesoamerican region. The term “management” involves all human activities transforming or maintaining nature in a given state according with a purpose or plan. Traditional plant management may include activities directly or indirectly favoring abundance and/or diversity of plants, whereas modern management forms commonly favor systems with lower diversity. Traditional plant management may include (i) strategies and communitarian agreements designed to planning use of forest products, (ii) intentional clearing, burning or even irrigation of forests in order to favor abundance of particular plant species, (iii) vegetative propagation or planting of seeds of the desired species and/or reducing competition from non-useful plants [11,37,63]. Several authors analyzing forms of incipient management of plants have identified the following types of management: tolerance, protection, and promotion [17,22,64]. Individual plants with desirable traits to the humans that manage them can be tolerated in particular areas, promoted by dispersing their vegetative or sexual propagules, and protected from competitors or herbivores [27,35,64]. However, all these practices not only involve the intention of increasing numbers of desirable plant resources. Also, people look for increasing the better resources and this practices involve artificial selection favoring quality of the resources managed in a system.

According to González-Insuasti and Caballero [63], incipient management may be nonselective and selective and artificial selection is an indicator of the differential intensity of plant management. According to these authors, selective incipient management is directed to increase and maintain the availability of desirable phenotypes in a population, with a consequent reduction in the frequency of undesirable phenotypes. Such a process may therefore maintain or increase the availability of desirable resources and increasing their quality (according to human values). These authors concluded that plants are within a gradient of management intensity following a gradient of manipulation from simple gathering of useful plant products to nonselective incipient management, selective incipient management, occasionally *ex situ* cultivated plants, and permanently cultivated domesticated plants. Blancas et al. [37] considered that artificial selection may occur at different levels of intensity, and this aspect also confers differential intensities to plant management.

The type and intensity of artificial selection associated to the different management forms discussed above trigger a series of structural changes which may be part of what has been called domestication syndromes [6,16,18]. Such syndromes are not easily discernible in species at incipient stage of domestication [52], but trends and consequences of selection are measurable and therefore analyzable from different perspectives as discussed below. The characteristics of the domestication syndromes were proposed mainly based on studies of annual species from temperate areas [65]. However, hundreds of plant species domesticated throughout the world have different characteristics; therefore, a deeper analysis of domestication syndromes deserves a broader scope of human experiences and ecological contexts and evolutionary trends associated to these variable aspects.

Artificial selection acting on plant populations may determine morphological, physiological, reproductive, and genetic changes, leading to phenotypic and genotypic divergence between wild and managed populations; the desirable characteristics being conserved and promoted by management practices [36,40,54]. Examples of this process have been extensively documented in Mesoamerican annual plant species such as maize (*Zea mays*), common beans (*Phaseolus vulgaris*) [66] and *Phaseolus lunatus*[61]. Among perennial plant species, several members of the Cactaceae family (especially columnar cacti and prickly pears, whose fruits are consumed by local people) are among the most studied [17]. For instance, species of *Opuntia*[67] and columnar cacti such as *Stenocereus stellatus* (Pfeiffer) Riccob. [38], *S. pruinosus* (Otto) Buxb. [21], *Polaskia chichipe* (Gosselin) Backeberg [41], *P. chende* (Gosselin) Gibson & Horak [40], *Escontria chiotilla* (F.A.C. Weber) F. Buxb. [36], and *Myrtillocactus schenckii*[37] can be mentioned. Species such as *Leucaena esculenta* (Moc. et Sessé) Benth. subsp. *esculenta*[54], *Crescentia cujete* L. [68]*Pithecellobium dulce* Benth. [69], *Sideroxylon palmeri* (Rose) Pennington [34], *Chrysophyllum cainito*[70], *Byrsonima crassofolia*[42], and *Ceiba aesculifolia* (H.B. & K.) Britten & Baker. subsp. *parvifolia* (Rose) P.E. Gibbs & Semir [35] are among the most representative of Mesoamerican trees studied relating ethnobotanical information on their management with resulting morphological and genetic patterns. *Agave* species, such as *A. fourcroydes* and *A. angustifolia*[71], and some species of palms [72] have also been studied with such a perspective.

In the case of Cactaceae, studies of wild, managed in situ, and cultivated populations showed that their edible fruits are highly appreciated by local people of several regions of Mexico. Fruit size (smaller sizes usually being more frequent in the wild whereas larger sizes are more frequent in cultivated populations), taste (sweeter fruit are more frequent in cultivated populations), thorniness (plants of wild populations are thornier), and mesocarp color (mainly red pulp in wild populations and other colors being more frequent in cultivated populations) are the main characteristics under selection [21,36-38]. Phenotypes producing fruit with the most desirable attributes according to local people are cultivated, which represents the highest level of artificial selection intensity. In the managed *in situ* or silviculturally managed populations the wild individuals showing the best attributes are let standing and enhanced and this artificial selection is relatively less intense than that practiced in cultivated populations.

*Leucaena esculenta* (Fabaceae) is another tree species studied in the context of incipient domestication. The number of seeds (higher amounts in those cultivated and managed in situ), the size of seeds and pods (larger in those cultivated and managed in situ than in the wild) are the variations that are most relevant to the morphological differentiation of wild populations, those tolerated in situ, and those that are cultivated. Also, flavor of seeds was identified as a relevant characteristic for local people. In this case “sweeter” flavor and digestible seeds are preferred over the indigestible and bitter ones. The phenotypic patterns found in cultivated and tolerated populations included traits that were more desirable compared to traits in wild populations [17,54,73].

The reproductive biology of some species has been studied hypothesizing changes in breeding systems associated to human management. Studies in several species of columnar cacti revealed that in most of them either wild and managed populations have self-incompatible breeding systems, indicating that in those cases artificial selection has not altered their breeding system [27,74]. However, in species such as *Polaskia chichipe*[41] and *Myrtillocactus schenckii*[39], self-compatibility occurs in wild populations and is significantly more frequent in silviculturally managed and cultivated populations. In addition, different animal species visit flowers of wild and managed populations, and periods of blooming peak may also differ among populations. Therefore, in addition to artificial selection, the reproduction systems may also help to explain morphological and genetic differentiation of wild and managed populations [27,39,41,75].

Human manipulation of natural resources not always decreases genetic diversity [76]. Studies evaluating the effects of human selection on genetic variation of plant populations were conducted in species, such as *Polaskia chichipe*[77], *Escontria chiotilla*[78], and *P. chende*[79]. In general, these studies have concluded that there is a slight reduction in genetic variation of silviculturally managed and cultivated populations when compared with wild populations. However, the opposite was recorded for *Stenocereus stellatus*[76] and *S. pruinosus* (Otto) Buxb. [21], in which some in situ managed and cultivated populations averaged higher genetic diversity than wild populations. One explanation to this increased diversity proposed by the authors is the continuous replacement of individuals in plantations, as well as the inclusion of types of these species from other villages. Furthermore, the authors also argued that tolerance and caring for seedlings and juveniles as well as seed dispersal by humans and animals appeared to contribute to the maintenance of local genetic diversity.

In general, the methods used for characterizing the patterns of domestication conducted in Mesoamerica, are helpful in the analysis of general patterns of plant domestication, since the selection associated with handling provides similar "measurable" results that allow researchers to visualize and investigate the human cultural causes of management and artificial selection on plants and their results.

### Brazilian cases

Even though studies on domestication of Brazilian plant species using ethnobotanical and evolutionary approaches are scarcer than in Mesoamerica, studies in the Amazon region have documented that fruit trees include a large number of species under different degrees of domestication, especially at incipient stages [11]. Out of all the species that have been identified as domesticated in the region, 27% are fruit-, nut-, and pod-producing species, while 87% of semi-domesticated species are represented by tree and vine species, and approximately 45 species the in incipient stage of domestication are almost all arboreal or chestnut trees [28]. According to Clements et al. [11], from the perspective of domestication, the more studied plant species in the Amazon region are *Manihot esculenta* Crantz. (cassava), *Theobroma cacao* L. (cocoa), *Ananas comosus* L. Merr. (pineapple), *Bactris gasipaes* Kunth. (Peach palm), *Paullinia cupana* Kunth. (Guaraná), *Capsicum* sp. (hot pepper), *Inga edulis* Mart. (inga), *Bertholletia excelsa* Bonpl. (Brazilian chestnut tree), and *Theobroma grandiflorum* (Willd. ex Spreng.) K. Schum (cupuaçú).

Another important case study is that on *Spondias tuberosa* Arruda which is pioneering in some study methods. *S. tuberosa* is a tree species native to the tropical dry forest called caatinga [15,80]. Our studies found that individuals of *S. tuberosa* are undergoing the process of incipient domestication. This conclusion is based on the fact that the *S. tuberosa* specimens are unintentionally and intentionally selected [80], and that the selection of targeted characteristics, when added to environmental variables and genetic variation, has resulted in phenotypic differences and divergence in fruit characters. Fruits can be found in various sizes and flavors in both managed and unmanaged areas, but in managed areas the fruits are significantly larger and tastier [15,80]. People maintain local phenotypic diversity in the fruit of *S. tuberosa* of different landscape units. Levels of genetic diversity are also well maintained in managed populations [81], which allowed to concluded that the local management practice of tolerance is strongly related to conservation of both morphological and genetic diversity of this plant species. In the southern region of Brazil, Santos et al. [14] studied the use and management of *Acca sellowiana* (O. Berg), finding phenotypic differences mainly in shape and color of the fruit between wild and managed populations and concluded that this species is in incipient domestication [82].

The studies referred to above are those that have started in Brazil documenting the use and management of plant species from the perspective of incipient domestication. However, due to the ecosystem, biological and cultural diversity of Brazil, certainly the application of methods for studying domestication of plants developed in Mesoamerica may potentially show interesting points in common and those that are particularly different.

Tables 1 and 2 summarize information from some of the main studies on incipient domestication conducted in Mexico and Brazil. In the perennial plant species, most of the examples found in Table 1, with respect to reproductive parts show the predominant trend of the selection in favor of larger and tastier fruits [83]. This pattern observed in Mexico as well as in Brazil (Table 2), also occurs in species of other regions of the World [83], indicating these as general selection targets and evolutionary trend of domestication of edible fruit trees [83]. However, it should be noted that for trees such as *Crescentia* spp. and *Ceiba aesculifolia* whose fruits are used as bowls and for fiber and edible seeds, respectively, shape and thickness of pericarp are similarly important as size [35,68].

**Table 1:** Examples of Mexican plants under domestication and their documented trends in changes resulting from artificial selection

**Species (Family)**	**Common name**	**Life form**	**Plant part used**	**Character**	**Selection trend**	**References**
*Agave spp.* (Agavaceae)	Sisal	Perennial herb	Fibers	Plant size	Larger Greater	[71,84]
				Leave’s	length and width	
				Teeth	Less abundant	
				Plant’s size and vigor	Greater	
		Pulque	Sap			
*Annanas comosus* L. merr. (Bromeliaceae)	Pineapple	c	Aggregated fruit	Seeds	Seedless fruits	[85]
				Taste, juiciness, color	Increasing sweet-tasting	
*Bactris gasipaes* (Arecaeae)	Pejibaye	Palm	Palm heart,	Fruit size	Increasing fruit size	[11,85-87]
			Fruit	Water content	Less	
				Firmness	Less	
				Pulp fibrousness	Less	
				Spines	Spineless or spines. Peach palms with spines used because their high-yielding and reasonably disease resistant	
*Byrsonima crassifolia* (L.) Kunth (Malpighiaceae)	Nance	Perennial tree	Fruits	Fruit size	Larger fruits	[42]
				Pulp flavor	Sweeter	
				Seed weigh	Lighter	
*Jacaratia mexicana* A. DC. (Caricaceae)	Bonete	Tree	Fruit	Size	Larger	[88,89]
				Pulp flavor	Sweeter	
				Pulp quantity	Greater	
*Opuntia ficus-indica* (L.) Mill (Cactaceae)	Prickly pear	Cacti	Cladode (stem)	Spines	Less abundant	[89-92]
				Thickness	Less	
				Mucilage	Less abundant	
				Vessel fibrosity	Less	
				Oxidation rate	Less	
				Size	Smaller	
				Glochids	Absent	
				Fruit	Smaller, less sweet	
			Fruit			
			(tuna)	Cladode size		
				Fruiting period	Larger Earlier in the year Younge	
				Reproductive age Glochids	Abundant	
				Seeds	Abundant	
			Fruit (xoconostle)			
				Fruit size	Larger	
				Peel/pulp rate	Higher	
*Pachyrhizus erosus* L. (Fabaceae)	Yam bean	Herbaceous vine	Tuberous root	Peel thickness	Reduce peel thickness	[93]
				Peel color	Favoring dark and white peel	
				Tuberous root size	Increasing tuber size	
*Persea americana* L. (Lauraceae)	Avocado	Tree	Fruit	Size	Larger Thicker (var. Hass)	[85,94]
				Peel		
*Sechium edule* Sw. (Cucurbitaceae)	Chayote	Vine	Fruit	Pulp flavor	Less sour	[95]
				Fibrosity	Less	
				Germination	In the tree (viviparism)	
*Spondias purpurea L.* (Anacardiaceae)	Jocote	Tree	Fruit	Size	Larger Other than red	[96]
				Color	Less	
				Sourness	More abundant	
				Pulp		
*Vanilla planifolia Jack ex Andr.* (Orchidaceae)	Vanilla	Vine, perennial climbing herb	Pods	Productivity	Higher flowering thus a major production of pods	[97]

**Table 2:** Examples of Brazilian plants under domestication and their documented trends in changes resulting from artificial selection

**Species (Family)**	**Common name**	**Life form**	**Plant part used**	**Character**	**Selection trend**	**References**
*Acca selowiana (O. Berg) Burret* (Myrtaceae)	Goiabinha serrana	Tree	Fruit	Fruit weight	Heavy fruit	[14]
				Length-diameter ratio	Elongated fruit	
				Fruit shape		
*Araucaria angustifolia* (Bertol.) Kuntze (Araucariaceae)	Pinheiro-Brasileiro	Tree	Pine	Pine size	Larger pine	[98]
				Productivity	More productive specimens	
				Pine flavor	Sweeter pine	
*Manihot esculenta ssp. esculenta* (Euphorbiaceae)	Cassava	Shrub	Tuberous root (sometimes leaves)	Stem cuts	Lower degree of branching favoring propagation by cuttings and lowering flowering, partial loss of defenses. Faster growth through change in seedling morphology	[99-101]
				Seedlings		
*Spondias tuberosa* Arruda (Anacardiaceae)	Umbuzeiro	Tree	Fruit	Fruit size	Larger fruit	[14,78]
				Pit size	Small pit	
				Fruit rind thickness	Thinner	
				Pulp yield	Higher yield	
				Fruit shape	Oblong fruit	
*Theobroma cacao* L.(Malvaceae)	Cocoa	Tree	Seeds	Fruit’s peel	Thinner (Pentagona type)	[102]
				Seed/fruit rate	Higher (Pentagona type)	
				Pulp flavor	Sweet (var. Criollo)	
				Fermentation time	Less (var. Criollo)	
*Euterpe oleraceae* Mart. (Arecaceae)	Açaí	Tree	Fruit	Fruit color	Purplish fruit	[89]
				Fruit flavor	Multiplos caules	
				Ramificação do caule		
*Bertholletia excelsea* Bonpl. (Lecythidaceae)	Castanheira	Tree	Fruit and seeds	Seed/fruit rate	Higher	[89]
*Theobroma grandiflorum* (Wild. ex. Spreng.) Schum (Malvaceae)	Cupuaçu	Tree	Fruit	Fruit size	Larger fruit	[89]
				Number of fruit	Higher	[89]
*Solanum sessiliflorum* Dunal (Solanaceae)	Cubiu	Perennial herb	Fruit	Fruit size	Larger fruit	
				Number of loculus	Firm fruits	
				Number of fruit	Higher	

Considering the biological and cultural diversity of Brazil, studies on plant management and domestication should be intensified. The Mesoamerican methods and models may be helpful for constructing a Brazilian framework to understand the dynamics of domestication guided by local Brazilian peoples. The increasing number of ethnobotanical studies conducted in the Northeastern region of Brazil, allows a favorable scenario to understand the processes of domestication of plants in semiarid areas as well as in the Amazon.

## Ethnobotany and its role in conservation of genetic resources

Studies during the 1970s evaluated the morphological variation among wild relatives and domesticated plants and focused on the deepening of morphometric intraspecific analyses of populations with different management histories [103]. Previously, morphological variations were evaluated among cultivated and wild relatives to address where the variations originated and why the process of domestication began. Since the 1970s, the main interest shifted to the process of domestication itself, focusing the attention on how domestication occurs [103].

From the 1980s, there has been an increasing number of studies concerning the genetic variation of plant populations under different management forms [103]. In the 1990s, ethnobotany developed a close interaction with evolutionary genetics and ecology, allowing considerable advances to understand the process of domestication. In such a context, ethnobotany has a crucial role to play for understanding the constellation of cultural aspects, motives and mechanisms of artificial selection and managed gene flow [21] put in practice by peoples to determine domestication of species and landscapes according to their constellation of purposes.

Ethnobotanical studies of incipient domestication in Mesoamerica have focused mainly to analyze domestication as an ongoing process [17,34,37,38,40]. These studies try to answer questions such as what are the targets of artificial selection in a species? How does the local cultural, economic and ecological factors influence the processes of domestication? What types of species are recognized locally? How are they perceived? Which are preferred and why? What are the main management practices locally used to direct artificial selection and gene flow? How different management forms determine different intensities of artificial selection? In this way, ethnobotany seeks to elucidate aspects related to the domestication as a holistic socio-ecological or bio-cultural process. The following questions are also priorities in further studies: What makes a plant likely to be chosen among other plants with similar potential use? Why to invest effort in managing a species but not in others? There may be numerous motives influencing how the choice is directed; therefore studies focused on these issues are imperious, as stated by Cleveland et al. [104]. Nevertheless, we must highlight that such decision-making by selection agents is crucial, not only to improve our understanding of the process of domestication, but also because it is helpful to identify main potential resources, priorities for conservation issues and local solutions developed to decrease risk in those important plant resources.

In few years, ethnobotany has developed and improved its methodological framework which is now a valuable body of tools for testing hypotheses and developing theories to elucidate questions about interactions between people and plants [105]. Interaction of ethnobotany with ecology, evolutionary genetics, and archaeology is nowadays a reality that has generated a research approach to understand the evolution of plants under domestication. Comparing patterns of domestication with similar methods provides the opportunity to understand general and particular contextual factors influencing domestication of species and landscapes of peoples of the World. In the New World it is particularly important to conduct deeper analyses comparing processes now occurring in main centers of origin of agriculture such as Mesoamerica, the Andean region of Peru, Bolivia, Argentina and Ecuador, as well as regions exceptional because of their high biological and cultural diversity, as are the Brazilian Amazonia and the semi-arid caatinga.

## Concluding remarks

Domestication of plants is an evolutionary continuous ‘biocultural’ [23] process. It is a process involving nature and society and should be therefore studied through holistic approaches. Ethnobotany has played an important role documenting the main cultural and biological factors influencing artificial selection and other evolutionary processes guided by humans to domesticate species and landscapes in territories. Processes of domestication are alive throughout the world and understanding how currently operate is crucial to analyze factors that in the past conducted to the origin of agriculture. But also, these studies provide key information for sustainable management of genetic resources for the future. The Mesoamerican methods and frameworks developed to analyze domestication are similarly applicable to understand the processes occurring in Brazil and vice versa. Therefore implementing research using similar methods should be emphasized in further studies in order to produce comparable information to understand general patterns of domestication.

## Competing interest

The authors declare that they have no competing interest.

## Authors’ contributions

All authors contributed with writing of the manuscript. All authors read and approved the final manuscript.
